# A microfluidic-based analysis of 3D macrophage migration after stimulation by *Mycobacterium*, *Salmonella* and *Escherichia*

**DOI:** 10.1186/s12866-022-02623-w

**Published:** 2022-08-31

**Authors:** Sandra Pérez-Rodríguez, Carlos Borau, José Manuel García-Aznar, Jesús Gonzalo-Asensio

**Affiliations:** 1 Department of Mechanical Engineering, Multiscale in Mechanical and Biological Engineering, University of Zaragoza, 50018 Zaragoza, Spain; 2 Aragon Institute of Engineering Research, University of Zaragoza, 50018 Zaragoza, Spain; 3 Grupo de Genética de Micobacterias. Departamento de Microbiología. Facultad de Medicina, Universidad de Zaragoza, IIS Aragón, 50009 Zaragoza, Spain; 4 CIBER Enfermedades Respiratorias, Instituto de Salud Carlos III, 28029 Madrid, Spain; 5 Instituto de Biocomputación y Física de Sistemas Complejos (BIFI), 50018 Zaragoza, Spain

**Keywords:** Microfluidics, Intracellular pathogens, Phagocytes, Chemotaxis, *Mycobacterium*, *Salmonella*, *Escherichia*, Respiratory pathogen, Diarrhea

## Abstract

Macrophages play an essential role in the process of recognition and containment of microbial infections. These immune cells are recruited to infectious sites to reach and phagocytose pathogens. Specifically, in this article, bacteria from the genus *Mycobacterium*, *Salmonella* and *Escherichia*, were selected to study the directional macrophage movement towards different bacterial fractions. We recreated a three-dimensional environment in a microfluidic device, using a collagen-based hydrogel that simulates the mechanical microarchitecture associated to the Extra Cellular Matrix (ECM). First, we showed that macrophage migration is affected by the collagen concentration of their environment, migrating greater distances at higher velocities with decreasing collagen concentrations. To recreate the infectious microenvironment, macrophages were exposed to lateral gradients of bacterial fractions obtained from the intracellular pathogens *M. tuberculosis* and *S. typhimurium*. Our results showed that macrophages migrated directionally, and in a concentration-dependent manner, towards the sites where bacterial fractions are located, suggesting the presence of attractants molecules in all the samples. We confirmed that purified *M. tuberculosis* antigens, as ESAT-6 and CFP-10, stimulated macrophage recruitment in our device. Finally, we also observed that macrophages migrate towards fractions from non-pathogenic bacteria, such as *M. smegmatis* and *Escherichia coli*. In conclusion, our microfluidic device is a useful tool which opens new perspectives to study the recognition of specific antigens by innate immune cells.

**Supplementary Information:**

The online version contains supplementary material available at 10.1186/s12866-022-02623-w.

## Introduction

The latest World Health Organization (WHO) report indicates that lower respiratory infections and diarrheal diseases remain one of the top 10 causes of death globally, and people living in low-income countries are far more likely to die from these diseases [1]. Both diseases are frequently caused by microorganisms. Among respiratory infections, Tuberculosis, which is mainly caused by *Mycobacterium tuberculosis*, remains today as the deadliest bacterial disease accounting for more than 1.5 million deaths in 2020, having higher prevalence in low-income countries. Tuberculosis is aggravated by the alarming emergence of drug resistant strains, and co-infection with Human Immunodeficiency Virus [2]. On the other hand, more than 420,000 people die annually from foodborne pathogens, with bacteria such as *Salmonella typhimurium* or *Escherichia coli*, being the most typical causative agents. These pathogens contaminate spoiled food which, when ingested, lead to the development of diarrheal diseases. These infections lead to more than half of the global deaths from foodborne pathogens, reaching 550 million affected and 230,000 deaths [3].

*M. tuberculosis* enters the body via the respiratory tract where it is engulfed by alveolar macrophages. Once phagocytized, *M. tuberculosis* is able to survive inside phagocytic cells. The most characteristic feature of this bacterium is its specialized secretion system, termed type seven secretion system (T7SS), or ESX, which releases different proteins [4]. Of the five ESX encoded in its genome, the most characterized is ESX-1, which releases two immunogenic proteins of *M. tuberculosis*, ESAT-6 and CFP-10. Both proteins form a dimer which enable phagosomal rupture, and the subsequent release of *M. tuberculosis* into the macrophage cytosol [5]. This cytosolic access facilitates the spread of *M. tuberculosis* to neighboring cells and the process of infection evolves resulting in pulmonary dysfunction [6].

*E. coli* and *S. typhimurium* are gram-negative bacteria belonging to the phylogenetic family of *Enterobacteriaceae*. *E. coli* usually coexist as a commensal in the gut, but some bacterial strains that have acquired virulent characteristics, become pathogenic [7]. These acquired virulence factors, are similar to those presented by pathogenic *Salmonella* species and include the secretion of toxins capable of killing eukaryotic cells, adhesion systems based on fimbriae or pili that allow bacteria to interact with host cells and/or the incorporation of flagella that enable bacterial motility [8]. Both bacteria also share the type III secretion system (T3SS), encoded in pathogenicity islands (PAIs), although each has unique machineries [9]. *E. coli* uses the T3SS to translocate effector proteins into host cells and control their signaling pathways, altering them to create a microenvironment to thrive. However, *S. typhimurium* has developed more sophisticated T3SS effectors to invade and replicate into intestinal epithelial cells and to survive within macrophage phagolysosomes. As a result of these virulent determinants, pathogenic *Salmonella* and *Escherichia* produce cell death and release of pro-inflammatory molecules that ultimately cause clinical diarrhea, among other diseases [10]. Both, in the lungs and in the intestine, there are innate immune cells, such as macrophages, responsible for containing bacterial infections. Macrophages possess a series of receptors that allow them to sense their environment and recognize the presence of pathogens. These receptors interact with the pathogens themselves or their secreted products [11], and activate a series of signaling cascades that generate changes in the cytoskeleton, so that the macrophage migrates towards the invading microorganism and is able to phagocytize it [12].

Therefore, since the outcome of the infection is strongly determined by the interplay between macrophages and bacteria, in this article we have focused on the study of the macrophage migration towards different bacterial stimuli. For this purpose, we have used 3D cultures on microfluidic devices. This technique allows working with microscale fluids [13] and is being increasingly used in the field of microbiology, because it offers numerous advantages such as better control of study conditions, biocompatibility of materials or cost and time savings. However, the most remarkable characteristic of this technology is the great versatility in terms of design that microfluidic devices offer. In addition, since most cellular infection studies to date has been performed using 2D cellular cultures in monolayers, they fail to recreate the 3D positioning of cells in a physiological environment, and they only allow to gain information about cellular movements in the *x* and *y* planes. Thus, using a microfluidic device in which cells are embedded within a hydrogel, allows cells to freely move and/or grow in the *x*, *y* and *z* planes, providing a full spatial dimensionality to the study [14].

Previous studies have studied macrophages-bacteria interactions using microfluidic devices. Some studies used microfluidic-based sorting devices to analyze cell populations of macrophages infected with *Francisella tularensis* or stimulated with *E. coli* LPS [15, 16]. However, they do not focus on the direct interaction between macrophages and bacteria. Other devices are designed to trap and isolate macrophages to study single-cell interactions with *E. coli* [17, 18], missing the information provided by the intercellular communication. In those studies in which macrophages and bacteria are co-cultured to take into account this cross-talk, they are routinely performed in liquid medium [19, 20], a physiologically unrealistic environment. Finally, the aforementioned studies have been predominantly performed with non-pathogenic bacteria. To our knowledge, there are limited studies addressing the interactions between macrophages and pathogens in microfluidic devices. Han et al*.* used a droplet-based microfluidic device coupled to electrodes to separate and isolate mixtures of macrophages and *S. typhimurium*, without analyzing the interaction between both cells. On the other hand, Gopalakrishnan et al*.* used a two-reservoir device connected by a network of microchannels to study individual movements of macrophages from one reservoir to the other containing bacteria from the *Mycobacterium avium* complex [21].

In this manuscript, we propose an alternative application of a previously described three-channel microfluidic design [22, 23], where macrophages are embedded in a collagen gel in the central channel and stimuli are applied from one side channel. This design presents a number of additional features not used before in the study of macrophage-bacteria interaction that overcomes the limitations of previous studies. First, the use of a collagen hydrogel offers three-dimensionality and mimics the extracellular matrix through which these innate cells must migrate to interact with pathogens. Second, by seeding a range of 100–500 cells per chip, we ensure that intercellular communication occurs. Third, side channels allow the generation of gradients by adding different conditions to each channel [24]. Fourth, the use of PDMS as the main material of the chip allows real time visualization of the events that are happening. Overall, our design allowed us to quantify the directional migration of macrophages to assess whether they were attracted to different fractions from representative bacteria in realistic microenvironment conditions.

## Methods

### Cell culture

We used THP-1 monocytes from the American Type Cell Culture (ATCC) that came from peripheral blood of one year infant human. THP-1 were cultured in suspension in RMPI-1640 supplemented with L-glutamine, 2% FBS and Ampicillin/Streptomycin. For monocyte differentiation to macrophages, THP-1 were resuspended at 6.5 × 10^5^ cell/ml in RPMI medium and 100 ng/ml phorbol 12-myristate-13-acetate (PMA) for 24 h. Macrophage became adherent, facilitating their distinction from undifferentiated monocytes.

### Bacteria culture and fractions preparation

We used these bacterial strains: *M. tuberculosis H37Rv* [25], *M. smegmatis* mc^2^155 [26]*, Salmonella enterica serovar typhimurium SV5015* [27] and *E. coli* DH5α. *M. tuberculosis* and *M. smegmatis* were grown in Middlebrook 7H9 broth supplemented with 10% (vol/vol) of ADC (0.5% bovine serum albumin, 0.2% dextrose, 0.085% NaCl and 0.0003% beef catalase) and 0.05% (vol/vol) Tween-80. *E. coli* and *S. typhimurium* was grown in Luria–Bertani (LB) broth.

Four bacterial fractions were prepared for the migration assays: secreted protein, bacteria inactivated by paraformaldehyde (PFA) treatment, bacterial lysate and bacteria inactivated by heat. For their preparation, bacteria were grown at a concentration of 10^9^ cfu/mL and then separated in four tubes used for the different treatments. To obtain the secreted protein, a trichloroacetic acid (TCA) precipitation was performed. Briefly, the sample was centrifuged and the bacterial pellet was discarded. The supernatant was incubated with 10% TCA for 1 h on ice and centrifuged. The pellet containing secreted proteins was washed in acetone, and the pellet was resuspended in distilled water. To inactivate bacteria by PFA treatment, the bacterial pellet was washed twice with PBS to remove extracellular components, resuspended in 4% PFA, and incubated for 1 h at room temperature. To obtain bacterial lysates, bacterial pellets were washed twice with PBS and then resuspended in 1 mL of cold PBS. Bacterial suspensions were disrupted by sonication using the BioRuptor (Diagenode) for 15 min (30 s pulse at high power), allowing cooling in an ice-water bath for 30 s between pulses. The samples were centrifuged at 4.000 g for 10 min at 4 °C, and the supernatant containing whole-cell bacterial extracts was filtered through a 0.22 μm-pore-size low protein-binding filter (Pall). For inactivating bacteria by heat treatment, bacterial pellets were washed twice with PBS, and then incubated at 100 °C for 10 min.

### Manufacturing of microfluidic device

Microfluidic devices were fabricated following the protocol published by Shin et al*.* in 2012 [28]. A polydimethylsiloxane (PDMS) mixture made of the base and curing agent of Sylgard 184 silicone elastomer in a 10:1 ratio was poured onto the printed wafer and incubated for 24 h at 80 °C. Then, PDMS molds were cut independently, perforated and autoclaved. Finally, both PDMS devices and 35 mm diameter glass bottom plates were treated with plasma to achieve a good seal between them. To increase the adherence of PDMS to the collagen hydrogel which is subsequently to be introduced, devices were filled with 1 mg/ml Poly-D-lysine (PDL) for 4 h at 37 °C and the washed with sterile water.

### Migration assays

A suspension of THP-1, at a final concentration of 3 × 10^5^ cell/ml, was embedded in collagen hydrogels. These hydrogels were made of a predefined concentration of type I collagen, to obtain the desired matrix stiffness, and an optimal NaOH/H_2_O balance to achieve a pH of 7. By working with the most abundant protein of the extracellular matrix and a physiological pH [29], it was possible to realistically recreate the extracellular environment in which macrophages migrate.

**Fig. 1:**
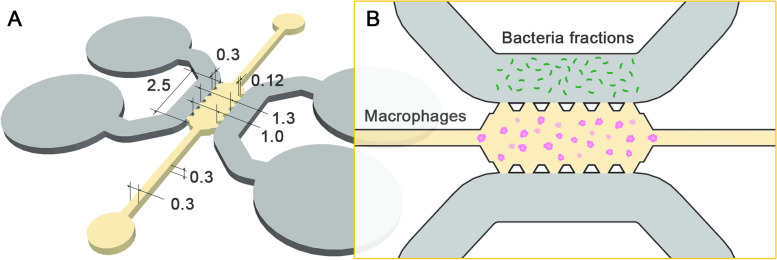
**A** Dimensioned top view of the microfluidic device consisting of a central channel (yellow) and two side channels (gray), each with corresponding loading ports at the ends of the channel. Dimensions are provided in millimeters. **B** Schematic drawing of the migration experiment design. Macrophages (pink) are embedded in a collagen gel in the central channel, while a gradient with bacterial fractions (green) is generated in the upper side channel

This hydrogel was introduced into the central channel of the microfluidic device (Fig. 1) and polymerized at 37 °C for at least 20 min in a humid box. Devices were turned around every 5 min to avoid adhesions between the cells and the PDMS or glass surface and to prevent them from passing into a two-dimensional plane. Subsequently, the reservoirs and side channels were filled with RPMI culture medium and the devices were incubated 24 h at 37 °C to let the macrophages to adapt to their three-dimensional environment before performing the migration assay.

Next day, the medium in the reservoirs was removed and the reservoirs were refilled differentially. In the case of the upper reservoir and channel, a 10^–4^ dilution of each bacterial fraction in RPMI medium was added, and the lower channel was filled with RMPI medium. In this way, a gradient was generated where the stimulus came from the upper part of the device. The devices were visualized in a phase contrast microscopy (Nikon D-Eclipse C1 Confocal Microscope, Nikon Instruments, Tokyo, Japan), which included an incubation chamber to maintain optimal culture conditions, taking pictures every 20 min for 24 h with 10 × objective lenses.

Between 5 and 9 devices were analyzed for each condition, and about 30–60 cells are tracked for each device, making a total of 150–350 cells analyzed per condition (Table S1). These data indicate that the present study is an exploratory research, in which the priority has been to expand the number of bacterial species and conditions, in order to show the behavior of macrophages under 21 different situations.

### Cell tracking and analysis

The focal plane located in the middle of the device along the z-axis was selected, and out-of-focus cells were not quantified to minimize artifacts resulting from the glass and PDMS surfaces, ensuring that the tracked cells were fully in a 3D environment. Cells were recorded for a total time of 24 h, taking images every 20 min, resulting in 72 images per chip.

Subsequently, individual cell migration was analyzed with a custom cell tracker algorithm developed in Matlab (Mathworks, Natick, CA, USA) [23] used in previous works [24, 30]. This software uses averaged pixel intensity information in search windows around each individual cell, therefore allowing centroid tracking at sub-pixel resolution. Besides, it allows visual correction from the user and post-process the migration results. In particular, trajectories were used to extract the cell mean (V_mean_, defined as the averaged instantaneous speed including all time steps) and effective (V_eff_, takes only into account the initial and final positions) velocities. The MSD curve of each trajectory was also obtained and used to determine the global diffusion coefficient (D) [31, 32], which was used as a measure of macrophage motility and migration persistence in a linear-weighted fit of the mean MSD curve (using the first quarter of the data). Additionally, MSD individual curves were used to fit a power law (MSD(t) = γ.t^α)) to determine the kind of motion (α < 1 for confined motion, α = 1 for Brownian or purely diffusive motion and α > 1 for directed motion).

Analysis of variance (ANOVA) followed by post hoc Tukey–Kramer tests were performed to determine statistical significance among the aforementioned parameters in the different conditions.

### Titration assays

Titration assays were performed under the same protocol as the migration assays: macrophages at a concentration of 3 × 10^5^ cell/ml were embedded in 2.5 mg/ml collagen gels. Macrophage migration was documented for 24 h, taking pictures every 20 min with a phase contrast microscopy with 10 × objective lenses. In this case, in each assay the stimulus to be studied was the same, but at different concentrations. The fractions of secreted protein from *M. tuberculosis* and *S. typhimurium* were analyzed at serial dilutions (1:10) with respect to the original secreted protein fraction in the range of 10^–2^ to 10^–6^. Purified ESAT-6 and CFP-10 proteins were kindly provided by Lionex GmbH (catalog numbers LRP-0017.3 and LRP-0016.2, respectively). These recombinant proteins were also analyzed in titration assays at concentrations ranging from 10 µg/ml to 1 ng/ml.

### Western blot

*M. tuberculosis* H37Rv cultures were grown in 7H9 (Difco) 0.05% Tween 80 supplemented with 0.2% dextrose and 0.085% NaCl, in order to avoid albumin contamination in the secreted protein fraction. After 2–3 weeks incubation at 37 °C, cultures were pelleted by centrifugation. The supernatant containing secreted proteins was incubated with 10% trichloroacetic acid (TCA) for one hour in ice and then centrifuged at 4 °C for 30 min. Pelleted proteins were rinsed with cold acetone and then resuspended in 150 mM TrisHCl pH 8. Protein integrity and absence of albumin contamination was checked by SDS-PAGE and Coomassie staining. Proteins were separated on SDS-PAGE 12–15% gels and transferred onto PVDF membranes using a semidry electrophoresis transfer apparatus (Bio-Rad). Membranes were incubated in TBS-T blocking buffer (25 mM Tris pH 7.5, 150 mM NaCl, 0.05% Tween 20) with 5% w/v skimmed milk powder for 30 min prior to overnight incubation with primary antibodies at the dilution indicated below. Membranes were washed in TBS-T three times, and then incubated with secondary antibodies for 1 h before washing. Antibodies were used at the following dilutions: mouse monoclonal anti-ESAT-6 antibodies at 1:1,000 for (abcam ab 26,246, HYB 076–08) and rabbit polyclonal anti-CFP10 antiserum at 1:2,000 (Thermo Scientific ref.: PA1-19,445). The corresponding horseradish peroxidase (HRP) conjugated IgG secondary antibodies (Sigma-Aldrich) were used at a 1:20,000 dilution. Signals were detected using chemiluminescent substrates (GE Healthcare).

## Results

### Macrophage migration and velocity are inversely proportional to collagen concentration in the microfluidic device

Macrophages are motile cells that migrate through the extracellular matrix (ECM) to sites of infection and inflammation. Since previous literature has demonstrated differences between cell migration in two-dimensional and three-dimensional environments [33], in this study, we recreated the extracellular matrix by confining a hydrogel of collagen I, in which macrophages were embedded three-dimensionally. In addition, we analyzed macrophage migration in gels of different collagen concentration, at 2.5, 4 and 6 mg/mL. These concentration changes imply structural changes in the hydrogel, such as smaller pore size or porosity, higher storage shear modulus and a decreasing hydraulic permeability with increasing collagen concentration [34].

**Fig. 2:**
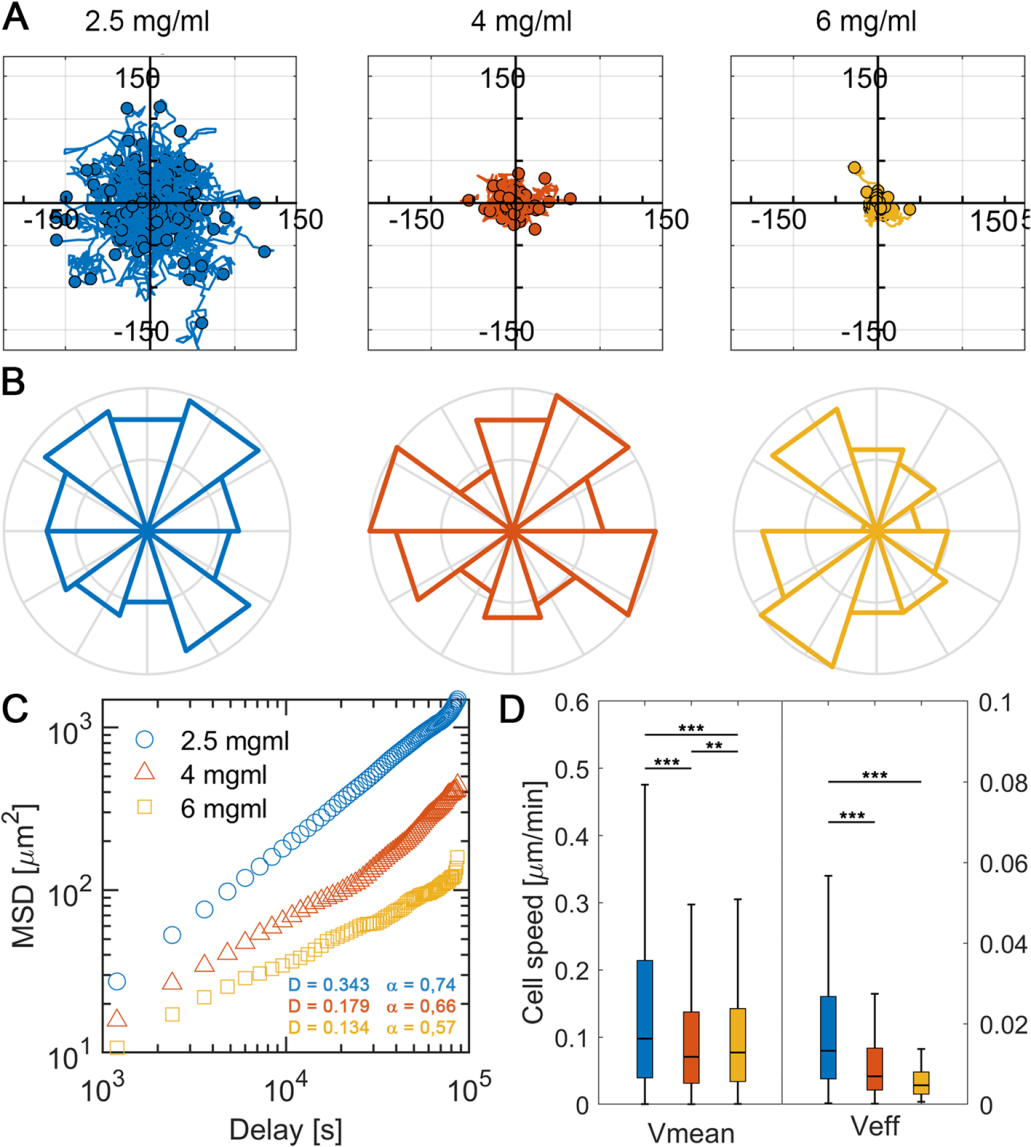
Macrophage migration in collagen gels of different concentrations: 2.5 mg/mL (blue), 4 mg/mL (orange) and 6 mg/mL (yellow). **A** Representation of the relative trajectories of macrophages, each line being the individual trajectory of a cell. **B** Directionality of migration considering the length of the radius as the number of cells that migrated in that direction and normalized by the total number of cells per condition. **C** Mean squared displacement (MSD) of the tracked trajectories, where D is the diffusive coefficient and α is the power law fitting coefficient (MSD(t) = γ.t^α). **D** Mean and effective velocity, where mean velocity is the total distance migrated divided by the time spent, and effective velocity is the distance between the starting and final point of the cell divided by the time spent. Asterisks indicate significant differences as follows: **p < 0.01, ***p < 0.005. n (devices) = 9 (2.5 mg/mL gels), 3 (4 mg/mL gels) and 3 (6 mg/mL gels), with an average of 25 cells per device

As the concentration of collagen in the hydrogels increases, macrophages migrate shorter total distances (Fig. 2A). In 2.5 mg/mL gels, some macrophages are able to overcome total distances of 100 μm from their point of origin, whereas as the concentration increases to 4 mg/mL, macrophages have more difficulty moving forward and their maximum migration distances are around 50 μm. In the case of the highest collagen concentration (6 mg/mL), macrophages do not reach distances greater than 25 μm. In all conditions, macrophages migrate radially without observing a directional movement, which is consistent with the absence of external stimulus (Fig. 2B). Quantitatively, no differences in terms of vertical migration were detected in the different collagen concentrations (Figure S1).

The analysis of the Mean Squared Displacement (MSD) of the macrophage trajectories provides information on the persistence of migration, reflected in the diffusive coefficient (D), and the type of movement that the cells have in this environment depending on the power law fitting coefficient (α) that they show (Fig. 2C). The increase in the concentration of collagen in the gels implies a decrease in the diffusive coefficient, indicating a lower persistence in the migration of macrophages. In 2.5 mg/mL gels, macrophages show a diffusive coefficient of 0.343 µm/min^2^, which decreases to 0.197 µm/min^2^ in 4 mg/mL gels, and to 0.134 µm/min^2^ in higher concentration gels at 6 mg/mL. In addition, in all the conditions an α value lower than 1 is obtained, indicating that in these assays macrophages showed a sub-diffusive (confined) type of movement, as opposed to a completely random walk behavior [35].

As the collagen concentration increases, the mean and effective velocities decrease significantly (Fig. 2D). However, comparing the obtained values of mean velocities with those published in previous literature, we observe that our values are clearly lower. In our collagen hydrogels, macrophages tend to migrate in a range of 0.05–0.2 μm/min, reaching velocities of 0.5 μm/min in the lowest concentration gels. In contrast, in vitro assays where macrophages migrated on 2D surfaces, velocities around 1 μm/min have been observed [36]. In vivo fish models, similar results have been obtained, with velocities in the range of 1–2.5 μm/min [37], which can increase over 10 μm/min in the presence of a wound [38]. For subsequent experiments with bacterial fractions we will work with 2.5 mg/mL collagen gels, because under these conditions they migrate longer distances and will allow us to follow cell trajectories more easily and with greater differentiation.

### Macrophages show directional migration towards bacterial fractions of *M. tuberculosis*

**Fig. 3:**
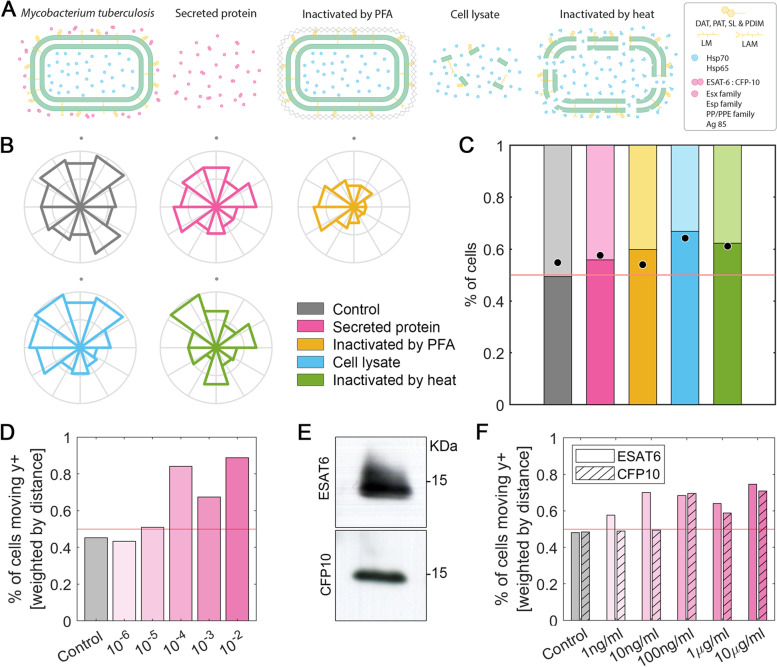
Macrophage facing *M. tuberculosis* stimuli. **A** Treatments applied to *M. tuberculosis* in order to obtain the different fractions subsequently tested. Intact bacteria consist of a mycobacterial membrane (green) with superficial components such as DAT, PAT, SL, PDIM, LM and LAM (yellow). Intracellular molecules like Hsp70 and Hsp65 are represented in blue, whereas extracellular secreted protein, like ESAT-6:CFP-10, Esp family, PP and PPE family and Ag85, are indicated in pink. Four treatments were performed to *M. tuberculosis*: secreted protein where only extracellular molecules were collected; inactivation by PFA that generates a crosslinking (grey mesh) preserving the superficial components; a cell lysate to break the cell and release superficial and intracellular components; and an inactivation by heat that damages the membrane (green) and denatures proteins (amorphous blue balls). **B** Migration of macrophages in 2.5 mg/ml collagen gels towards a gradient generated at the top (+ y axis, indicated by grey dots) with *M. tuberculosis* bacterial fractions: non-stimulated control (gray), secreted protein (pink), inactivated by PFA (yellow), cell lysate (blue) and inactivated by heat (green). Directionality of migration, where the radius is the number of cells migrating in each direction and normalized by the total number of cells per condition. **C** Percentage of macrophages as a function of the verticality of their migration and weighted by the total distance traveled. Those macrophages whose final position is above their initial position contribute to the bar graph exceeding the red line set at 0.5. The dots in each bar graph correspond to the percentage of macrophages whose final position is above or below their starting position, but not weighted by the total distance migrated. n (devices) = 9 (control), 8 (secreted protein), 6 (inactivated by PFA), 6 (cell lysate) and 5 (inactivated by heat). **D** *M. tuberculosis* secreted protein fraction titration assay. The y-axis shows the percentage of macrophages migrating in the y-plane weighted by the total distance moved; the x-axis shows the concentrations of serial dilutions (1:10), ranging from 10^–6^ to 10^–2^ (pink intensity), including the control without bacterial stimulus (gray). The red line set at 0.5. **E** Western blot of the secreted protein fraction of *M. tuberculosis* in which ESAT-6 and CFP-10 proteins were detected. Unprocessed and uncropped versions of the membranes can be visualized on Supplementary Figure S3. **F** Titration assay of purified ESAT-6 (smooth bar) and CFP-10 (striped bar) proteins. The y-axis shows the percentage of macrophages migrating in the y-plane weighted by the total distance moved; the x-axis shows the concentrations of serial dilutions (1:10), ranging from 1 ng/mL to 10 µg/mL (pink intensity), including the control without bacterial stimulus (gray). The red line set at 0.5

Once characterized the optimal conditions for macrophage migration in our microfluidic device, we prompted to simulate macrophage stimulation with *M. tuberculosis* bacterial fractions (Fig. 3A). Each fraction presents a different composition. On the one hand, the secreted protein exclusively contains those proteins released by the bacteria to the extracellular milieu. When bacteria are inactivated by PFA, a cross-linking reaction preserves bacterial surface macrostructures [39]. This implies that PFA-inactivated bacteria maintain their bacterial wall intact, so that macrophages will be able to interact with all the components of their surface. The cell lysate contains either the cytosolic components, or the membrane fractions from lysed bacteria, but not the secreted protein fraction. Finally, when bacteria are inactivated by heat, molecule denaturation occurs [40]. This leads to alterations in the bacterial cell wall, allowing release of cytosolic components. Therefore, this fraction contains denatured cell wall and cytosolic components.

A gradient was generated individually for each bacterial fraction in the microfluidic devices, and the macrophage migration was monitored for 24 h. Next, we quantified the number of macrophages that migrated in each direction from their point of origin, and represented this migration in a rosette plot (Fig. 3B). Since the gradient with bacterial stimulus is generated in the upper part of the devices, it provides qualitative information on whether the macrophages have migrated directionally towards these bacterial extracts. This qualitative information is transformed into quantitative information enumerating the percentage of macrophages whose final position was above or below their initial position, weighted by the effective distance traveled (Fig. 3C).

Qualitatively, when exposing macrophages to *M. tuberculosis* fractions, a clear directional migration toward the secreted protein, its cell lysate, and bacteria inactivated by heat treatment is observed, since the areas of the upper portions in the rosette plot are higher than in bottom portions. In contrast, in the non-stimulated control, macrophage migrate in the same proportion to the upper or the lower area of the microfluidic device (Fig. 3B). Analyzing the quantitative information, the randomness in the migration of macrophages without the presence of stimulus is demonstrated, since the percentage of cells migrating towards the top or the bottom of the hydrogel, with respect to their original position and weighted by the distance traveled, is similar. However, when generating a gradient with any of the *M. tuberculosis* fractions, the percentage of macrophages that migrate towards the top of the device, where the stimulus is located, always exceeds the 0.5 baseline. The fractions promoting the higher migration were the cell lysate and bacterial extracts inactivated by heat, inducing more than 60% macrophages effectively migrating towards the stimuli (Fig. 3C).

After determining that the different *M. tuberculosis* fractions exert an attractant effect on macrophages, we analyzed whether different concentration of the stimuli influenced macrophage migration. For this purpose, serial dilutions with respect to the original secreted protein fraction were studied. The results show that macrophages migrate attracted to the bacterial stimulus, regardless of the concentration of the fraction, as long as it exceeds a minimum threshold. In cases where the fraction is diluted to 10^–6^ and 10^–5^ concentrations, migration percentages in the y-plane are similar to the unstimulated control. In contrast, a clear peak in the verticality of migration is observed when moving from the 10^–5^ dilution to the 10^–4^ dilution. This could indicate that the sensitivity range of the assay for this study fraction is in the range of a 10^–4^ and 10^–5^ dilution. Subsequent more concentrated dilutions than 10^–4^ show high percentages of migration towards the top of the chip (Fig, 3D).

The secreted protein fraction contains an uncharacterized composition of molecules. However, numerous proteins secreted by *M. tuberculosis* are described in the literature. In particular, the two more immunogenic proteins from this pathogen are ESAT-6 and CFP-10 [41]. First, we confirmed the presence of both proteins in our secreted protein sample by western blot (Fig. 3E). Then, we performed titration assays with purified samples of ESAT-6 and CFP-10 proteins, in a range of concentrations from 1 ng/mL to 10 µg/mL. In the case of ESAT-6, the most diluted concentration, 1 ng/mL, was able to stimulate the directional migration of macrophages. A gradual increase in directionality of migration is observed as the stimulus concentration is increased from 1 to 10 ng/mL. However, once this concentration is reached, the percentage in vertical migration remains around 0.7. In the case of CFP-10, we again observed a threshold effect, in which concentrations of 1 and 10 ng/ml do not stimulate the directional migration of macrophages. However, by increasing the concentration to 100 ng/mL, we observed a prominent rise in verticality (Fig. 3F). Altogether, these results support the hypothesis of a minimum concentration threshold that must be exceeded to induce macrophage directional migration in our device, and this threshold depends on the specific stimulus.

### Macrophages show directional migration towards bacterial fractions of *S. typhimurium*

**Fig. 4:**
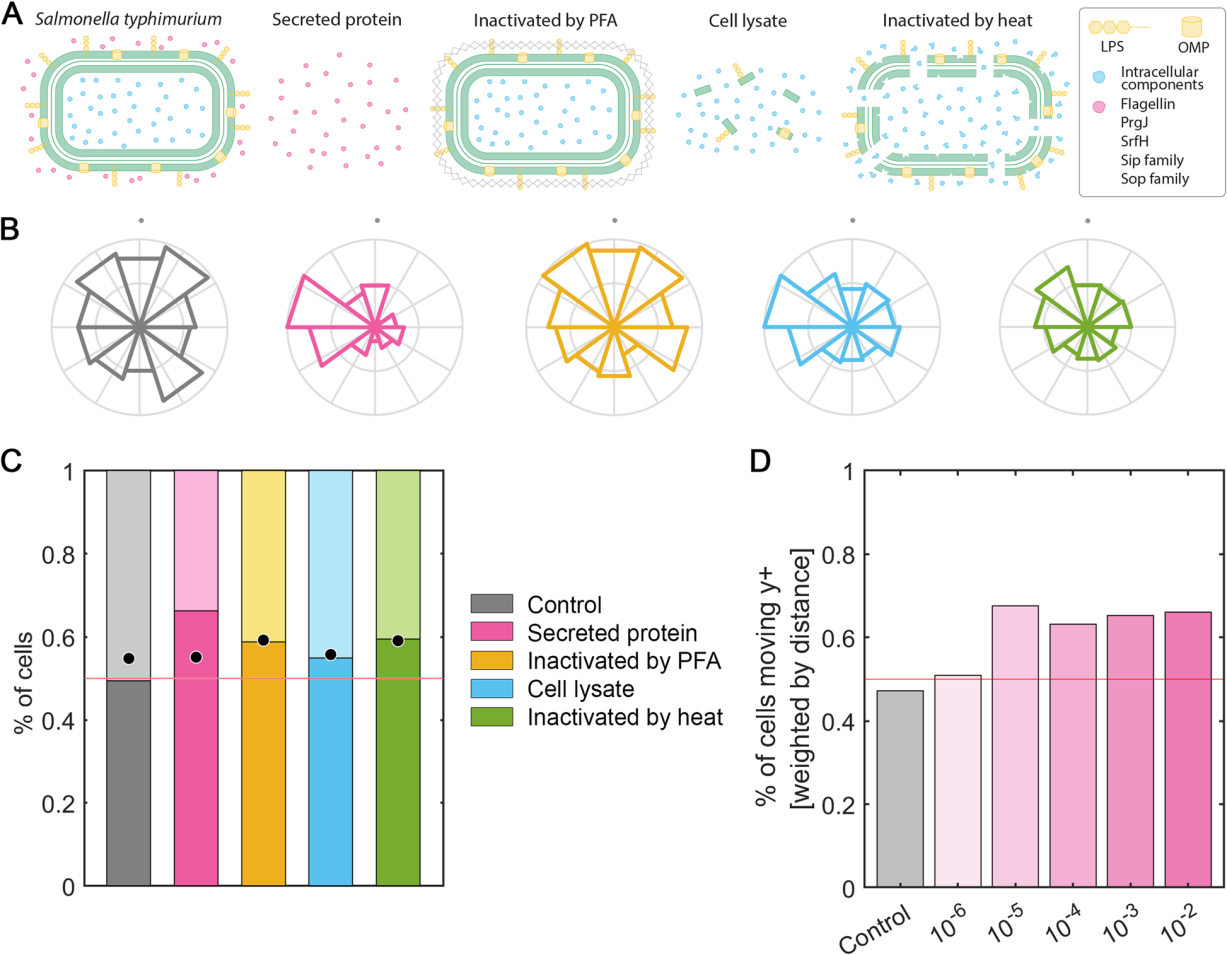
Macrophage facing *S. typhimurium* stimuli. **A** Treatments applied to *S. typhimurium* in order to obtain the different fractions subsequently tested. Intact bacteria consist of a gram-negative membrane (green) with superficial components such as LPS or OMP porines (yellow). Intracellular components are represented in blue, whereas extracellular secreted protein, like flagellin, PrgJ, SrfH, Sip and Sop family, are indicated in pink. Four treatments were performed to *S. typhimurium*: secreted protein where only extracellular molecules were collected; inactivation by PFA that generates a crosslinking (grey mesh) preserving the superficial components; a cell lysate to break the cell and release superficial and intracellular components; and an inactivation by heat that damages the membrane (green) and denatures proteins (amorphous blue balls). **B** Migration of macrophages in 2.5 mg/ml collagen gels towards a gradient generated at the top (+ y axis, indicated by grey dots) with *S. typhimurium* bacterial fractions: non-stimulated control (gray), secreted protein (pink), inactivated by PFA (yellow), cell lysate (blue) and inactivated by heat (green). Directionality of migration, where the radius is the number of cells migrating in each direction and normalized by the total number of cells per condition. **C** Percentage of macrophages as a function of the verticality of their migration and weighted by the total distance traveled. Those macrophages whose final position is above their initial position contribute to the bar graph exceeding the red line set at 0.5. The dots in each bar graph correspond to the percentage of macrophages whose final position is above or below their starting position, but not weighted by the total distance migrated. n (devices) = 9 (control), 6 (secreted protein), 5 (inactivated by PFA) and 5 (cell lysate) and 5 (inactivated by heat). **D** *S. typhimurium* secreted protein fraction titration assay. The y-axis shows the percentage of macrophages migrating in the y-plane weighted by the total distance moved; the x-axis shows the concentrations of serial dilutions (1:10), ranging from 10^–6^ to 10^–2^ (pink intensity), including the control without bacterial stimulus (gray). The red line set at 0.5

We applied those conditions tested in *M. tuberculosis* to *S. typhimurium*, since both bacteria are intracellular pathogens targeted by macrophages (Fig. 4A). Macrophage migration was analyzed in 2.5 mg/mL collagen gels for 24 h. Qualitatively, it is observed that macrophages preferentially migrate towards all *S. typhimurium* fractions (Fig. 4B). By weighting the number of macrophages migrating toward the gradients generated with the *Salmonella* fractions, it is demonstrated that all *S. typhimurium* fractions exert an effect on macrophages by attracting them directionally. Migration towards secreted protein results in the highest macrophage migration relative to the remaining fractions, exceeding 0.65 value (Fig. 4C). Titration assays with the secreted protein from *S. typhimurium* show the same migration pattern previously observed with *M. tuberculosis*. At concentrations below the threshold, (10^–6^ dilution relative to the fraction used in panels B and C), macrophages migration is similar to the non-stimulated control. As soon as the concentration is increased to a 10^–5^ dilution, macrophages migrate directionally towards the top of the hydrogel. Once reached the threshold, this verticality of migration is maintained more or less constant, regardless of the stimulus concentration applied (Fig. 4D).

### Macrophages migrate towards fractions of non-pathogenic *E. coli* and *M. smegmatis*

**Fig. 5:**
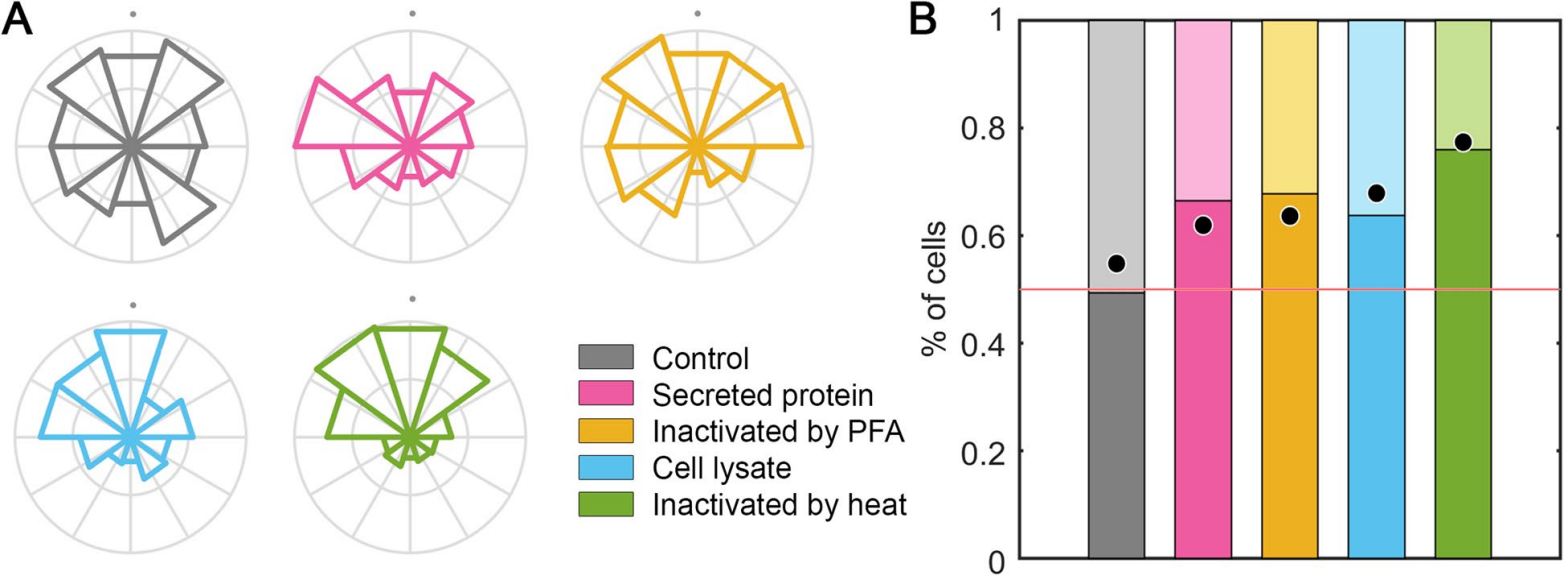
Migration of macrophages in 2.5 mg/ml collagen gels towards a gradient generated at the top (+ y axis, indicated by grey dots) with *M. smegmatis* bacterial fractions: non-stimulated control (gray), secreted protein (pink), inactivated by PFA (yellow), cell lysate (blue) and inactivated by heat (green). **A** Directionality of migration, where the radius is the number of cells migrating in each direction and normalized by the total number of cells per condition. **B** Percentage of macrophages as a function of the verticality of their migration and weighted by the total distance traveled. Those macrophages whose final position is above their initial position contribute to the bar graph exceeding the red line set at 0.5. The dots in each bar graph correspond to the percentage of macrophages whose final position is above or below their starting position, but not weighted by the total distance migrated. n (devices) = 9 (control), 5 (secreted protein), 5 (inactivated by PFA), 5 (cell lysate) and 5 (inactivated by heat)

Since macrophages are innate immune cells able to recognize foreign antigens, they are able to target invading microorganisms irrespective of their pathogenic or non-pathogenic nature. Accordingly, once demonstrated the migration of macrophages towards pathogenic bacterial fractions, we sought to demonstrate that these innate immune cells are also recruited in the presence non-pathogenic microorganisms. Migration assays were also performed in response to stimuli from *M. smegmatis*, a mycobacteria commonly used as a non-pathogenic model of *M. tuberculosis*. Both qualitatively and quantitatively, a clear directional attraction of macrophages upon exposure to all bacterial fractions of *M. smegmatis* was observed (Fig. 5A and B). Specifically, the fraction that recruits the higher percentage of macrophages is the heat-inactivated bacterium, with more than three quarters of the macrophages migrating towards it.

**Fig. 6:**
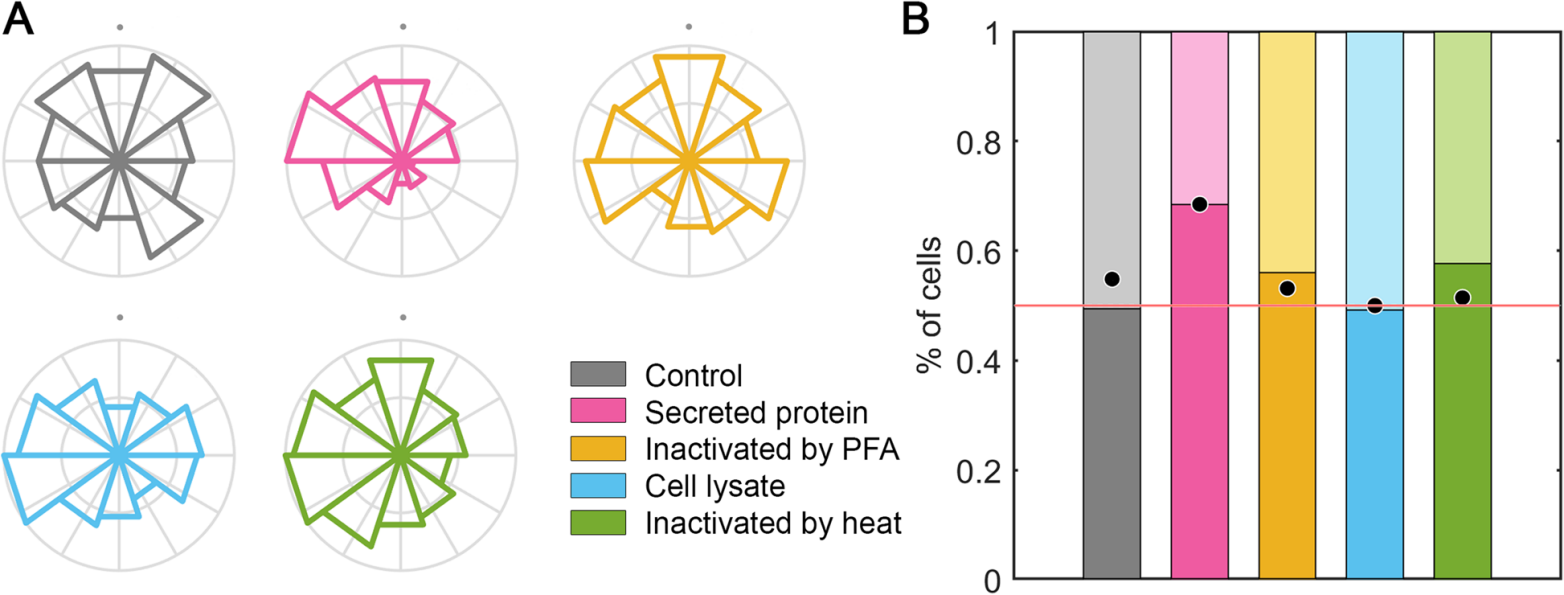
Migration of macrophages in 2.5 mg/ml collagen gels towards a gradient generated at the top (+ y axis, indicated by grey dots) with *E. coli* bacterial fractions: non-stimulated control (gray), secreted protein (pink), inactivated by PFA (yellow), cell lysate (blue) and inactivated by heat (green). **A** Directionality of migration, where the radius is the number of cells migrating in each direction and normalized by the total number of cells per condition. **B** Percentage of macrophages as a function of the verticality of their migration and weighted by the total distance traveled. Those macrophages whose final position is above their initial position contribute to the bar graph exceeding the red line set at 0.5. The dots in each bar graph correspond to the percentage of macrophages whose final position is above or below their starting position, but not weighted by the total distance migrated. n (devices) = 9 (control), 5 (secreted protein), 5 (inactivated by PFA), 5 (cell lysate) and 5 (inactivated by heat)

The response of macrophages to *E. coli DH5α* bacteria was also tested. This *E. coli* strain is a non-pathogenic bacterium, routinely used in laboratories as a genetic model. Qualitatively, macrophages preferentially migrate toward the secreted protein and the PFA-fixed bacteria (Fig. 6A). However, quantitatively, it is observed that macrophage, not only sense attraction to these two fractions, but also to bacteria inactivated by heat (Fig. 6B). Overall, the chemotactic response generated by the secreted protein fraction results in up to 70% macrophage recruitment. In contrast, the cell lysate failed to stimulate a clear directional migration of macrophages.

Even though *M. smegmatis* and *E. coli* fractions are able to recruit macrophages, it is key to remember that under a physiological environment, the fate of these bacteria within the macrophage differs from that of pathogenic *S. typhimurium* and *M. tuberculosis*. Specifically, intracellular multiplication of *M. smegmatis* and *E. coli DH5α* is restricted in activated macrophages, since these bacteria does not possess intracellular survival mechanisms, unlike their *M. tuberculosis* and *S. typhimurium* counterparts [42, 43].

## Discussion

Pathogen-host interaction has been studied by molecular- and cellular-based methods. However, despite having contributed invaluable knowledge, these methods still show difficulties in mimicking a physiological infection. For example, in vitro or ex vivo models do not include the incorporation of realistic flow or biomechanical environments and culture conditions are sometimes not suitable for the growth of all microorganisms. This leads to results obtained that do not always correlate with in vivo models [44]. The emergence of new technologies, such as microfluidics, allows overcoming some of these limitations [14]. Specifically, the microfluidic device used in this article allows us to generate a three-dimensional biomechanical environment that recreates the extracellular matrix through which macrophages physiologically migrate. This is of utmost importance, since it has been shown that the two-dimensional and three-dimensional migration of this cell type presents differences, such as greater variation in cell dynamics and morphology in 3D environments [45].

Our three-dimensional approach presents some limitations that have to be carefully analyzed to understand the impact of our research work. First, although macrophages are fully embedded in a three-dimensional hydrogel, their movement in the z-plane is limited by the central chamber height of 300 microns. Second, when analyzing their migration, a single z-stack is chosen, so although the movement is three-dimensional, only the cells located in this plane are tracked. Finally, the cells present a small random motion around themselves, which has been defined as morphodynamics. These changes in cell shape contribute to migratory motion [46], but can give rise to abnormally high mean velocities, since this measure encompasses cell positions of all the frames, averaging the instantaneous velocities between them. Therefore, even small vibrations will contribute to the final value. To avoid the misinterpretation of these results, especially in analyses with small cell sizes and short migrated distances, mean speeds are shown together with effective velocities, that only take into account the initial and final position of the cells.

For providing directional migration, a stimulus must first be present, as demonstrated by the lack of macrophage directional movement in the non-stimulated controls. Subsequently, this stimulus is sensed and a signaling cascade is generated that activates the cytoskeleton machinery so that the macrophage migrates towards the received signal [47]. Although there are numerous stimuli of different nature in cell migration, such as chemical or mechanical [48], the current literature on macrophage migration has only described stimuli of biochemical origin. This implies that a physical contact must be established between the signaling molecule and the sensitive macrophage receptor [49]. The design of the microfluidic device used in this study allows the generation of a chemical gradient from the side channel where the molecules of interest are introduced to the central chamber where the three-dimensional cell culture is located [50]. The diffusive coefficient of a particle in a hydrogel is defined by the Stokes–Einstein equation modified to take into account the porosity of the matrix (not to confuse with the diffusive term D of the MSD analysis that represents macrophage motility and movement persistence). Thus, the effective diffusion coefficient of a particle (*D*_*p*_) is calculated following the formula:

$${D}_{p}=\frac{{k}_{B}T}{6\pi \eta r}\cdot \mathrm{exp}\left(-\left[\sqrt{\varphi }\cdot \left(1+\frac{r}{{r}_{f}}\right)\right]\right)$$

where k_B_ is Boltzmann's constant, T is the absolute temperature, η is the viscosity of the fluid, r is the radius of the diffusing molecule, *φ* is the void ratio of the matrix and r_f_ is the radius of the fiber [50]. Thus, we hypothesize that the components of the bacterial fractions diffuse into the hydrogel until they come into contact with macrophages.

The bacterial fractions used in the present study consist on a great variety of molecules of different sizes, which makes it impossible to calculate the diffusion coefficient of each individual component. However, the size of the largest component, which is the intact bacterium after fixation with PFA, is known. Typically, a prokaryotic bacteria has an average length of 1–5 microns. Therefore, for the calculation of the effective diffusive coefficient we are going to use as study molecule a particle of 3 microns in diameter. The fractions are dissolved in RPMI medium supplemented with 10% fetal bovine serum, which assumes a dynamic viscosity of the medium of approximately 0.95 mPa x s [51]. Migration assays are carried out on 2.5 mg/mL collagen gels, whose three-dimensional structure has been studied by Olivares et al*.* determining that the void ratio is 85.01% and the approximate radius of the collagen fibers is 0.12 microns [52]. Applying these data to the previously described formula, we obtain an effective diffusion coefficient of 1.77—10–14 m^2^ x s^−1^. This indicates that the whole bacterium is able to diffuse through the hydrogel and will come into contact with the macrophages, stimulating them. Therefore, we hypothesize that if the largest component of the fractions is able to diffuse, the remaining molecules, which are smaller in size, will be also able to diffuse towards the central channel containing cells. This would result in the overall trend of macrophages to migrate upwards when bacterial fractions are present, exceeding in all conditions the migration parameters of the non-stimulated control. However, despite the clear trend in directional migration when bacterial fractions are present, statistical differences were not found among conditions (Supplementary Figure S2). This is probably imposed by the technical limitation in analyzing 13 experimental conditions using 95 chips, so that a low number of chips (5 to 9) were analyzed in each condition (See Table S1 for details). Since our study aims to establish a *proof-of-concept* to study bacterial migration using microfluidics, we have preferred to design wide, exploratory, analyses; but we are confident that focusing on a single experimental condition would have resulted in stronger statistical differences.

The *M. tuberculosis* fractions contains well-known molecules that interact with macrophages. This pathogen contains in its genome five specialized secretion systems, known as ESX1-5 [53]. Among them, the ESX-1, which secretes ESAT-6 and CFP-10 is finely characterized. These genes are found in the RD1 region, which is lacking in the BCG tuberculosis vaccine, indicating their essentiality in the virulence of this bacterium [54]. ESAT-6 and CFP-10 each contains a dense repertoire of T-cell epitopes and are known for their immunogenicity; indeed, they are used as vaccine candidates [41]. Both proteins induce recruitment of macrophages to infected areas in in vivo models [55]. In line with these observations, we have demonstrated a concentration-dependent macrophage migration when using purified ESAT-6 and CFP-10 antigens. This result opens translational applications, since other relevant antigens, from any cell or microorganism, could be tested using our microfluidic design. This knowledge has a direct and useful application in the biomedical and vaccine field.

On the other hand, *S. typhimurium* is characterized by a series of virulence-associated genes that are divided into 17 pathogenicity islands (SPI) throughout its genome, with SPI-1 being the most important. The best-known virulence system of *Salmonella* is the type III secretion system (T3SS), which translocates effector proteins, both to the extracellular matrix and to the cytoplasm of host cells thanks to its needle-like structure [56]. Since these SPI are absent in *E. coli* DH5α, it is tempting to speculate that differences in macrophage migrations towards *Salmonella* and *Escherichia* fractions might be explained on the basis of differential antigen composition in both bacteria.

Furthermore, we determined that the directional migration of macrophages is regulated by the concentration of the bacterial stimulus. A migration model is proposed in which there is a threshold concentration that must be exceeded to induce the attraction of these immune cells. Once exceeded, macrophages show the same percentage of verticality in their movement, regardless of the concentration of the fraction.

Even if application of microfluidics to microbiology is an emerging field, recent studies show that this technique is gaining momentum. Kim et al*.* were able to grow intestinal microbiota bacteria and intracellular pathogens in a microfluidic device that recreated the gut. The composition of this device was based on the culture of a monolayer of intestinal epithelium on a hydrogel that recreates the ECM and the passage of fluid flow at low rate that mimics peristaltic movements [57, 58]. Thacker et al*.* used a similar, but more complex approach in which they co-cultured lung epithelium, along with endothelial cells and macrophages to recreate lung physiology. In this case, *M. tuberculosis* was introduced to mimic a realistic infection situation [59]. It should be also noted that the mechanical environment, such as stiffness, pH or hydrodynamics, also influences cell behavior [60]. Our microfluidic device would allow replacing the collagen-based matrix by other hydrogel options that bring new features to study macrophage motility characteristics. Scaffolds can be made sensitive to temperature, with polysaccharides such as hyaluronic acid or cellulose, to electric fields using sulfonated polystyrene, or to pH using polyacrylamide [61]. These perspectives show the great potential and versatility of the application of microfluidics in microbiology.

## Supplementary Information

**Additional file 1: Table S1.** Number of cells analyzed in migration assays. **Figure S1.** Vertical analysis of the migration of macrophages in gels of different collagen concentration. **Figure S2.** Statistical analysis of macrophage migration in y-plane towards bacterial stimuli. **Figure S3.** Original versions of the Western-blot membranes shown in Figure 3E.

## Data Availability

Raw and processed data about macrophage migration will be made available upon request to jmgaraz@unizar.es or jagonzal@unizar.es.
