# Supplementary material for: A microfluidic-based analysis of 3D macrophage migration after stimulation by Mycobacterium, Salmonella and Escherichia
Source: BMC Microbiol. 2022 Aug 31;22:211. doi: 10.1186/s12866-022-02623-w (PMC9429415; doi:10.1186/s12866-022-02623-w)
Supplement: Supplementary file 1 — Additional file 1: Table S1. Number of cells analyzed in migration assays. Figure S1. Vertical analysis of the migration of macrophages in gels of different collagen concentration. Figure S2. Statistical analysis of macrophage migration in y-plane towards bacterial stimuli. Figure S3. Original versions of the Western-blot membranes shown in Figure 3E. [file 12866_2022_2623_MOESM1_ESM.pdf]

SUPPLEMENTARY MATERIAL

Table S1. Number of cells analyzed in migration assays

CONTROL			
# cells per chip	total	condition	# chips
[28;40;35;54;60;49;38;22;26]	352	'Control'	9

M. tuberculosis			
# cells per chip	total	condition	# chips
[31;37;29;23;35;71;38;26]	290	'Secreted protein'	8
[32;27;47;34;35;25]	200	'Inactivated by PFA'	6
[34;34;30;39;47;34]	218	'Cell lysate'	6
[30;18;45;44;38]	175	'Inactivated by heat'	5

S. typhimurium			
# cells per chip	total	condition	# chips
[17;16;16;75;35;54]	213	'Secreted protein'	6
[28;24;37;24;61]	174	'Inactivated by PFA'	5
[37;36;25;39;46]	183	'Cell lysate'	5
[54;33;51;40;59]	237	'Inactivated by heat'	5

M. smegmatis			
# cells per chip	total	condition	# chips
[68;41;46;59;80]	294	'Secreted protein'	5
[61;107;48;79;54]	349	'Inactivated by PFA'	5
[82;40;45;42;43]	252	'Cell lysate'	5
[67;79;66;53;57]	322	'Inactivated by heat'	5

E. coli			
# cells per chip	total	condition	# chips
[11;11;10;42;70]	144	'Secreted protein'	5
[23;23;18;47;34]	145	'Inactivated by PFA'	5
[39;24;34;61;6]	164	'Cell lysate'	5
[28;32;29;69;56]	214	'Inactivated by heat'	5

Table S1. Number of cells tracked per chip, total cells and total chips according to bacterial species: M. tuberculosis; S. typhimurium; M. smegmatis and E. coli; and subdivided by study condition: unstimulated control (gray), secreted protein (pink), inactivated by PFA (yellow), cell lysate (blue) and inactivated by heat (green).

Figure S1. Vertical analysis of the migration of macrophages in gels of different collagen concentration.

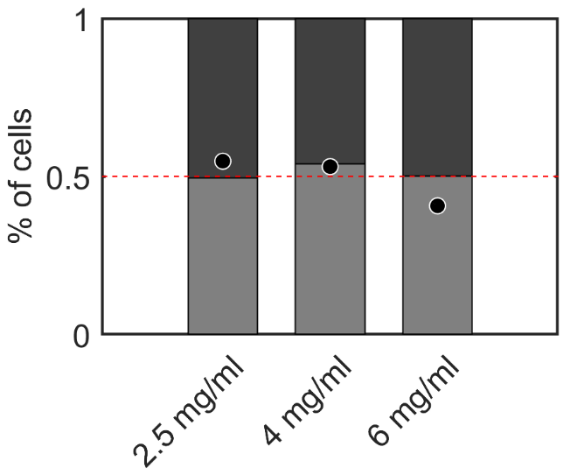

Fig S1. Vertical migration of macrophages in hydrogels of 2.5 mg/mL, 4 mg/mL and 6 mg/mL collagens. Bars indicate the percentage of macrophages migrating upwards (light grey) and downwards (dark grey) weighted by the total distance traveled. Red dashed line is set at 0.5. The black dots in each bar represent the percentage of macrophages whose final position is above or below their starting position regardless the total distance migrated (non-weighted). Overall, collagen concentration does not affect macrophages directional migration in the y-plane.

Figure S2. Statistical analysis of macrophage migration in y-plane towards bacterial stimuli.

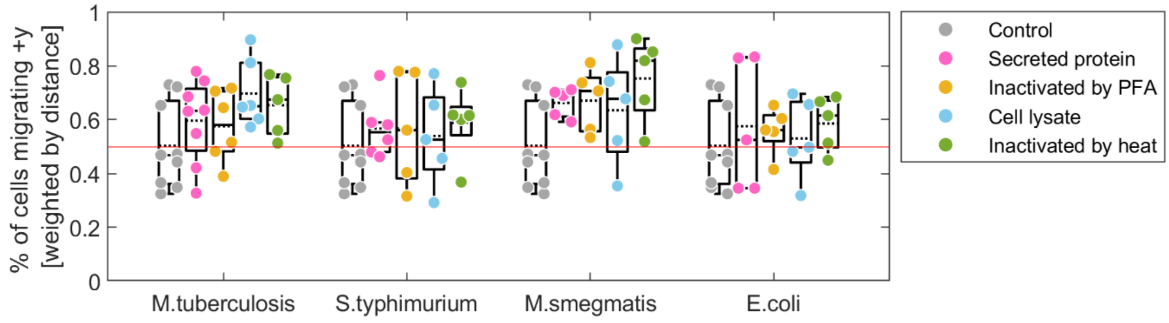

Figure S2. Percentage of cells migrating upwards (+y), weighted by the total distance traveled in the y-direction, in the different study conditions. Each point represents the final percentage per microfluidic device analyzed. Conditions are grouped by bacterial species: M. tuberculosis; S. typhimurium; M. smegmatis and E. coli; and subdivided by study condition: unstimulated control (gray), secreted protein (pink), inactivated by PFA (yellow), cell lysate (blue) and inactivated by heat (green). Data is represented by boxplots in which the dotted line represents the median and the dashed line the mean. Red line is set at 0.5. Note the trend to migrate towards the upper part of the y-axis,

exceeding in all conditions the median and mean of the control. No significant differences were found between study conditions after applying ANOVA and post-hoc tests.

Figure S3. Original versions of the Western-blot membranes shown in Figure 3E

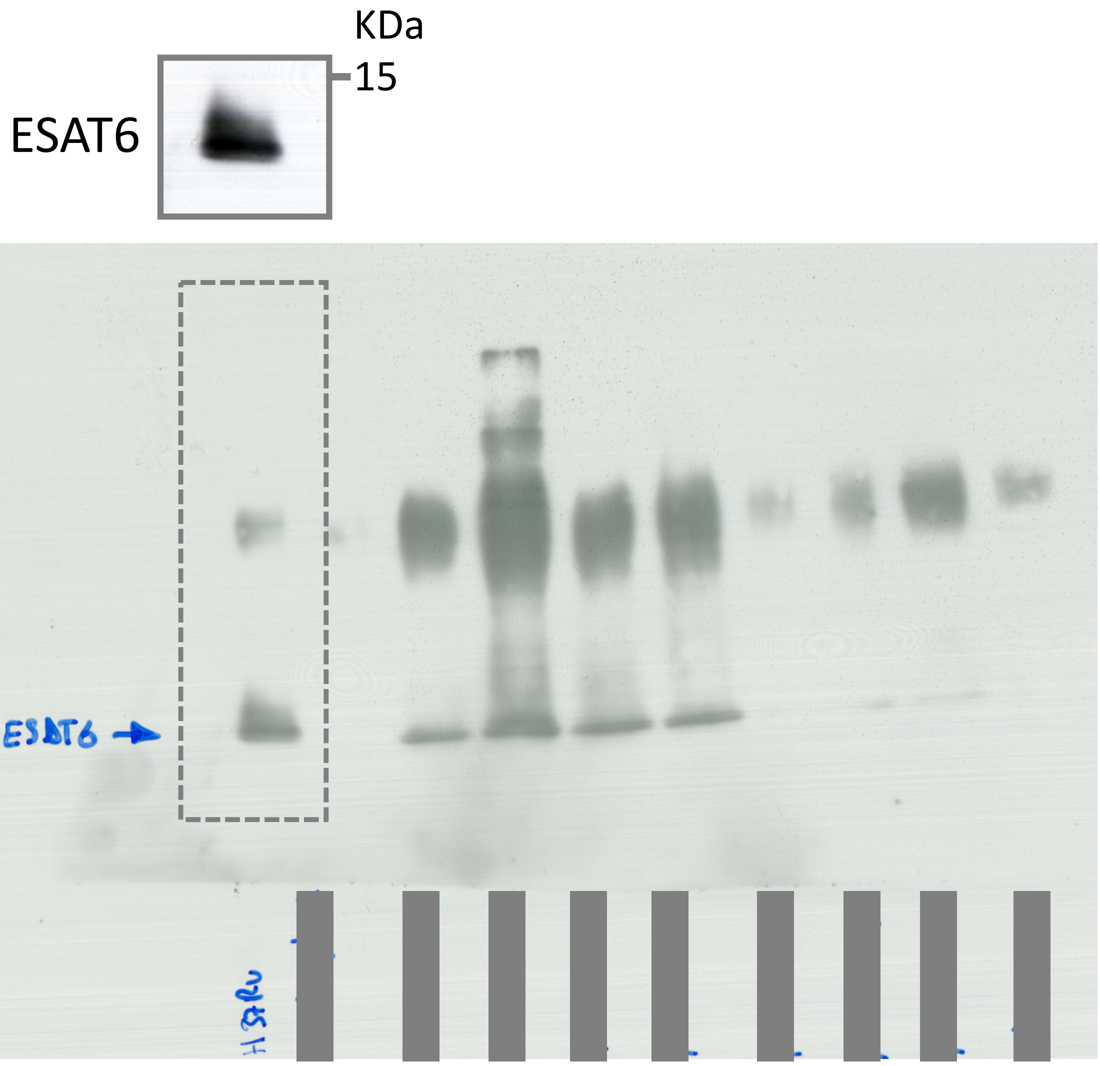

Full image

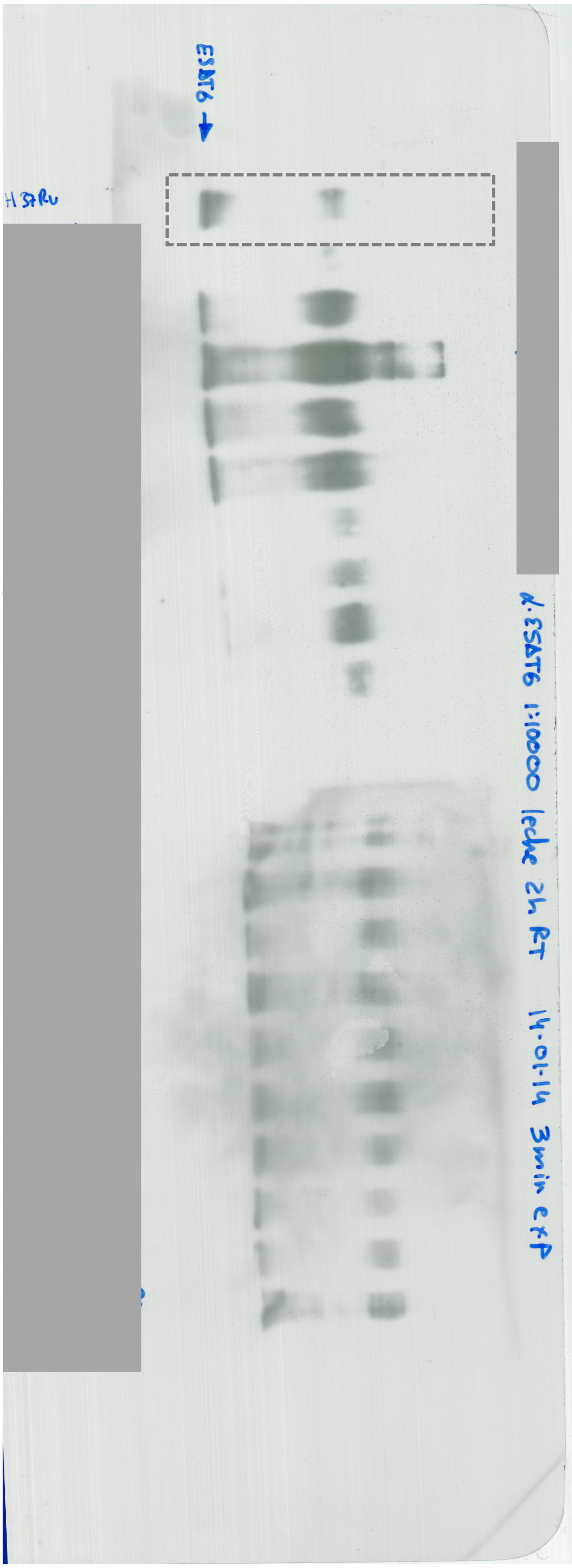

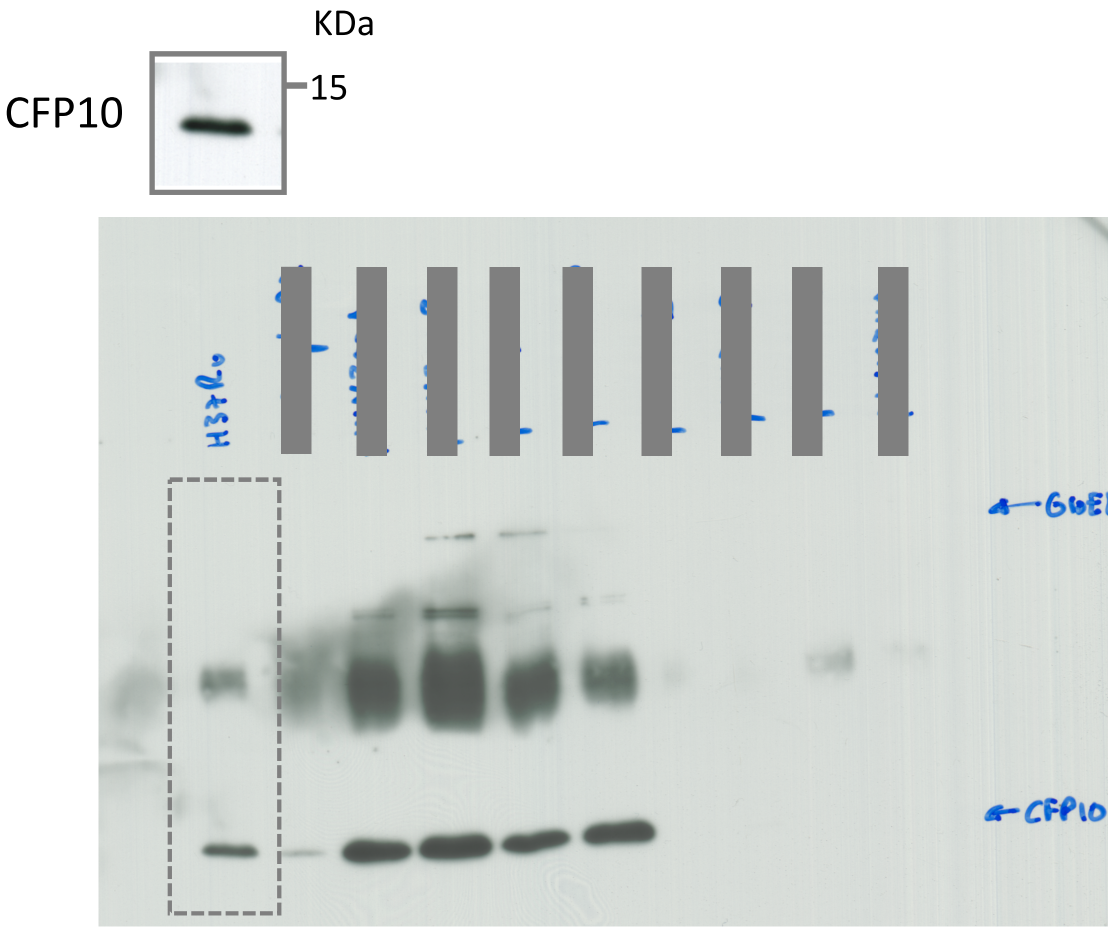
KDa
15
CFP10
CFP10

Full image

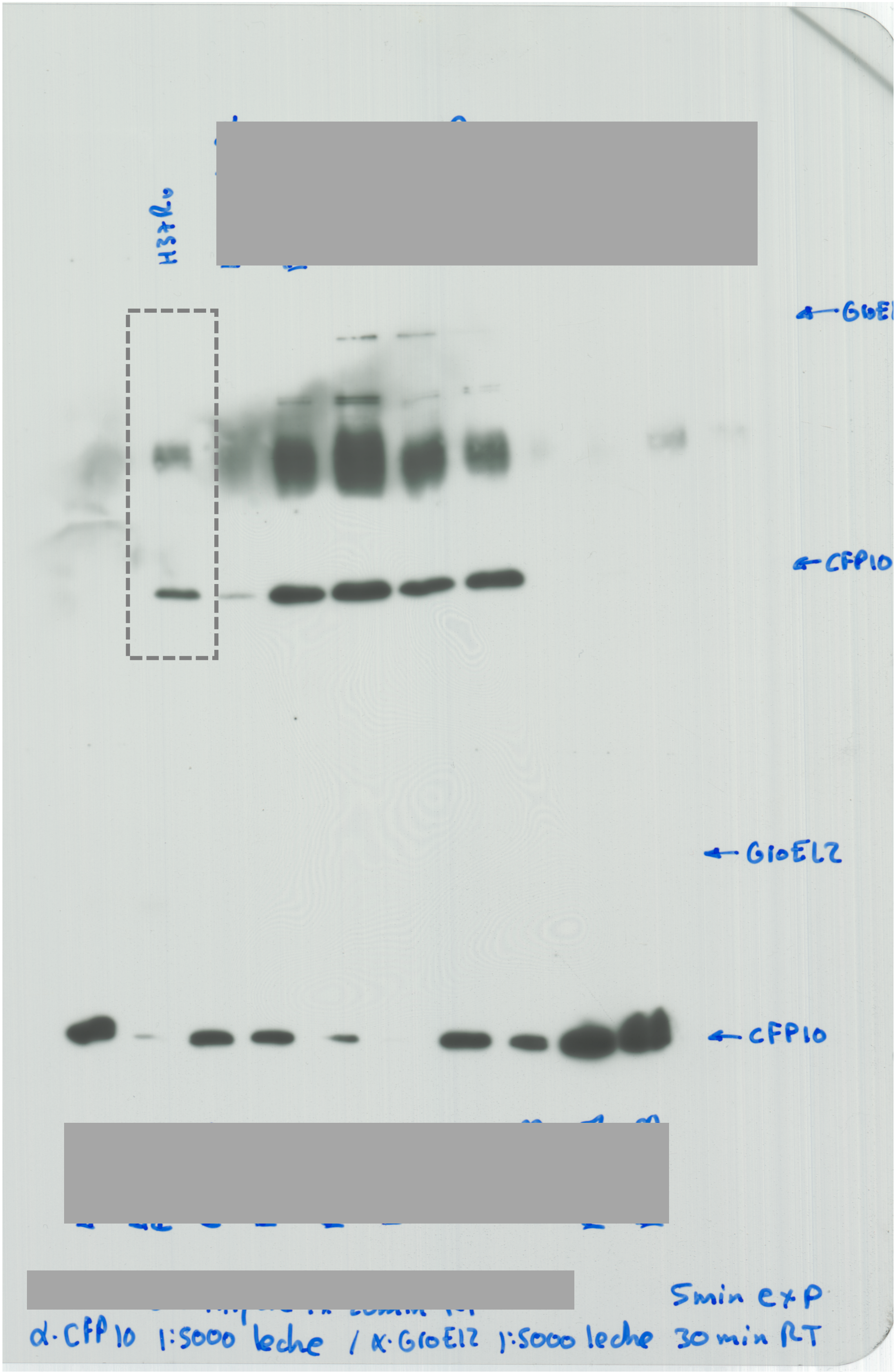

Figure S3. Original, unprocessed and uncropped scanned images from Western-blot membranes used for detection of ESAT-6 and CFP-10 proteins in Figure 3E. Note that

ESAT-6 and CFP-10 are low molecular weight proteins of 6 and 10 KDa, respectively. Lanes indicating the presence of ESAT-6 and CFP-10 in M. tuberculosis H37Rv used in the present study are indicated by a dashed line. Since gels were used for detection of ESAT-6 and CFP-10 for an independent study, the content of the remaining lanes has been covered for confidential purposes.
